# Análise biomecânica comparativa de duas técnicas de fixação da osteotomia do olécrano

**DOI:** 10.1055/s-0045-1811927

**Published:** 2025-11-18

**Authors:** Felipe Lacerda de Oliveira Pessôa, Marcio Liu Sandt, Marcos Alves Correia, Carlos Rodrigo de Mello Roesler, Maria Eugenia Leite Duarte, Verônica Fernandes Vianna

**Affiliations:** 1 Serviço de Ortopedia, Hospital Copa D'Or, Rede D'Or São Luiz, Rio de Janeiro, RJ, Brasil; 2 Laboratório de Engenharia Biomecânica, Hospital Universitário da Universidade Federal de Santa Catarina, Florianópolis, SC, Brasil; 3 Instituto D'Or de Pesquisa e Ensino (IDOR), Rio de Janeiro, RJ, Brasil

**Keywords:** biomecânica, dispositivos para fixação cirúrgica, fraturas do úmero, olécrano, osteotomia, biomechanic, humeral fractures, olecranon process, osteotomy, surgical fixation devices

## Abstract

**Objetivo:**

Comparar as características biomecânicas da fixação da osteotomia do olécrano utilizando parafusos transcorticais (TCs) ou intramedulares (IMs).

**Métodos:**

Ulnas sintéticas de poliuretano foram seccionadas simulando a osteotomia tipo Chevron. As osteotomias foram fixadas com parafusos TCs ( *n* = 11) ou IMs ( *n* = 11). Após a fixação, os corpos de prova foram montados em um dispositivo de posicionamento na máquina de ensaios e submetidos a pré-carga de 10 N, seguida por 100 ciclos de carregamento entre 10 e 500 N. Ao final dos 100 ciclos, a carga de 500 N foi mantida e a abertura do foco de fratura ( *gap* ) na região da osteotomia foi medida. A seguir, foi aplicado carregamento monotônico de tração até a falha da fixação e obtenção das medidas da força de resistência máxima, da rigidez dos sistemas e do modo de falha.

**Resultados:**

Nenhum dos grupos apresentou falha após a aplicação dos ciclos de carga entre 10 e 500 N, sem diferença nos valores do *gap* ( *p* = 0,9420). A força máxima de falha no grupo IM foi 1,27 vezes maior do que no grupo TC ( *p* = 0,0459) e a rigidez dos 2 sistemas foi semelhante ( *p* = 0,670).

**Conclusão:**

As duas técnicas mostraram ser alternativas eficazes em termos de estabilidade e rigidez. A fixação com parafuso intramedular demonstrou maior capacidade de suportar cargas antes da falha, sugerindo possível vantagem em termos de resistência mecânica. Os resultados do presente estudo poderão auxiliar na interpretação das implicações clínicas das duas técnicas nas investigações sobre métodos de fixação da osteotomia do olécrano.

## Abstract (en)

**Objective:**

To compare the biomechanical characteristics of olecranon osteotomy fixation using transcortical (TC) or intramedullary (IM) screws.

**Methods:**

We sectioned synthetic polyurethane ulnas to simulate a Chevron osteotomy. Osteotomy fixations were performed using TC ( *n* = 11) or IM screws ( *n* = 11). After fixation, we assembled the specimens on a positioning device in the testing machine to subject them to a preload of 10 N, followed by 100 loading cycles ranging from 10 to 500 N. At the end of the 100 cycles, we maintained the 500-N load and assessed the opening of the fracture focus (gap) in the osteotomy region. Then, we applied monotonic tensile loading until fixation failure and measured the maximum resistance force, system stiffness, and failure mode.

**Results:**

No group presented failure after the application of load cycles ranging from 10 to 500 N, with no difference in gap values ( *p* = 0.9420). The maximum failure force in the IM group was 1.27 times greater than in the TC group ( *p* = 0.0459). The stiffness of the 2 systems was similar ( *p* = 0.670).

**Conclusion:**

Both techniques were effective alternatives in terms of stability and rigidity. Fixation with IM screws resulted in better load-bearing capacity before failure, suggesting a potential advantage in mechanical strength. The results of the present study may help the interpretation of the clinical implications of the two techniques in investigations on fixation methods for olecranon osteotomy.

## Introdução

As fraturas do cotovelo correspondem a 7% das fraturas em adultos, com as fraturas distais do úmero representando menos da metade desse total. 1 Com um padrão de distribuição bimodal, essas fraturas ocorrem em homens jovens devido a traumas de alta energia e em mulheres idosas, em decorrência de fragilidade óssea. 2 Independentemente da faixa etária, o tratamento cirúrgico, embora desafiador devido à complexidade anatômica da articulação do cotovelo, é considerado o padrão-ouro. 1 3 O tratamento conservador é reservado para fraturas extra-articulares sem desvio, para pacientes com contra-indicações clínicas à cirurgia, para aqueles com deficit neurológico no membro e para aqueles com risco elevado de complicações locais. 1

As opções cirúrgicas incluem abordagens medial e lateral paratricipital, que preservam ou dividem o tríceps, além da osteotomia do olecrano, técnica preferida para a redução aberta e fixação interna das fraturas intra-articulares. 4 Esta abordagem oferece melhor acesso à fratura e uma visualização articular superior em comparação com técnicas que não utilizam osteotomia. 4 5 6 7 8

Diversos tipos de osteotomia já foram descritos, 9 sendo a abordagem posterior em “V” tipo Chevron a mais utilizada, por proporcionar uma visão abrangente da epífise distal do úmero e uma boa exposição intra-articular. 1 7 8 9 10 11 12 A técnica oferece maior estabilidade rotacional e favorece a consolidação e a estabilidade da fratura devido à ampla área de contato entre as superfícies ósseas. 4 8 No entanto, alguns estudos relatam complicações associadas às osteotomias, como pseudoartrose, perda da redução articular, falha na síntese e implantes sintomáticos, 12 13 14 15 o que tem motivado a busca por novas alternativas de fixação. 4 5 6 12

Historicamente, as bandas de tensão são a principal técnica de fixação das osteotomias, devido ao seu potencial para converter as forças de distração do tríceps em forças compressivas no local da fratura. 15 No entanto, estudos clínicos destacam as vantagens da fixação da osteotomia com parafusos intramedulares (IMs) 16 17 ou transcorticais (TCs). 7 10 A fixação IM envolve a inserção de um parafuso longitudinalmente no canal medular do olecrano, com mínima violação das partes moles. 16 17 Os menores índices de complicações associados à fixação IM são atribuídos à menor agressão local, à ausência de migração do implante 16 17 18 e à menor complexidade da técnica em comparação com as bandas de tensão ou placas e parafusos. 15

Outra alternativa para fixação da osteotomia do olecrano é a utilização de parafusos TC. 7 10 Nessa técnica, dois parafusos TC são inseridos perpendicularmente ao local da osteotomia, atravessando ambas as corticais. 19 A técnica é considerada mais simples do que a fixação IM e oferece resistência a esforços cisalhantes, o que pode ser uma vantagem no período inicial da reabilitação. 7

Embora as técnicas de fixação IM e TC sejam aplicáveis clinicamente para a fixação da osteotomia, não há consenso sobre o desempenho biomecânico de cada uma, especialmente em relação à estabilidade da fixação e à força máxima de resistência. O objetivo do presente estudo foi realizar uma análise biomecânica em ossos sintéticos, comparando a fixação da osteotomia do olécrano tipo Chevron utilizando parafuso IM ou parafusos TC. Os parâmetros de avaliação incluíram a resistência a cargas cíclicas, a força máxima para atingir a falha da fixação e a rigidez das montagens.

## Materiais e Métodos

### Ossos Compósitos

Foram utilizados vinte e dois ossos compósitos de ulna esquerda (Nacional Ossos, código 3020) e dois ossos compósitos radiopacos do mesmo fabricante (código 12333) para comparar as características biomecânicas de 2 sistemas de fixação da osteotomia com: (i) 1 parafuso IM ou (ii) 2 parafusos TCs.

### Materiais de Síntese e Grupos de Pesquisa

Foram utilizados parafusos canulados de rosca parcial com arruela (7,0 × 90 mm) e parafusos de rosca parcial com arruela (4,0 × 42 mm), ambos fornecidos pelo mesmo fabricante (Traumédica). As ulnas foram divididas aleatoriamente em 2 grupos de acordo com o método de fixação da osteotomia com 1 parafuso IM (grupo IM; *n* = 11) ou com 2 parafusos TCs (grupo TC; *n* = 11).

### Osteotomia

Antes da realização das osteotomias, foram feitas marcações para definir o eixo longo da ulna no local da inserção distal do tendão do tríceps, na porção proximal do olécrano e na região diafisária. A osteotomia foi realizada com serra oscilatória, simulando a osteotomia tipo Chevron. 8 Para definir a área a ser osteotomizada, foi aplicado um molde com angulação de 140°, com o ápice distal posicionado sobre o eixo longo previamente demarcado na ulna, 2 cm distal à superfície óssea proximal do olécrano. A osteotomia foi realizada com angulação de 20° no plano sagital.

### Fixação das Osteotomias

#### Parafuso Intramedular

**Fig. 1:**
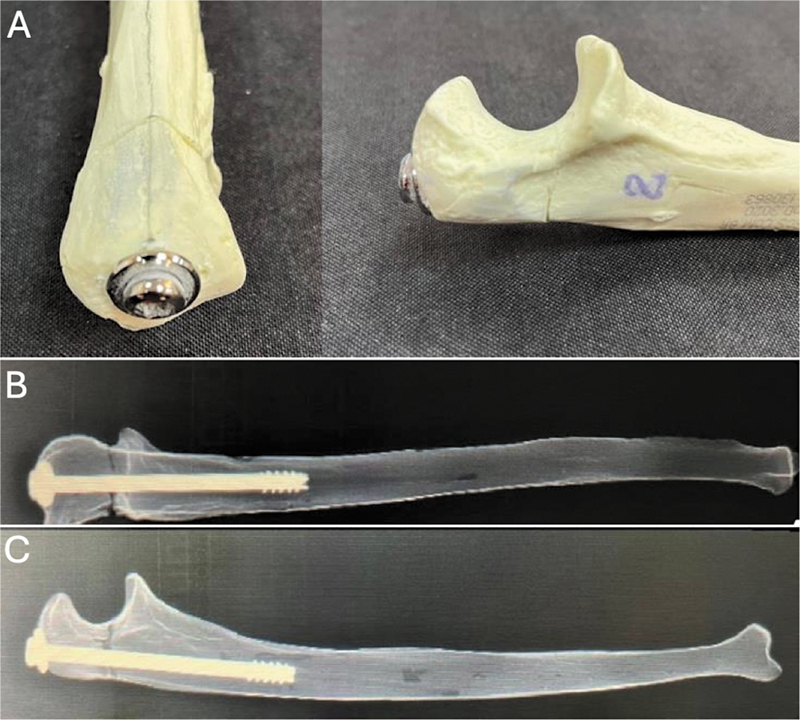
Fixação com parafuso intramedular (IM). (A) Fixação da osteotomia com a inserção longitudinal de um parafuso canulado no canal medular. (B) Radiografias nas incidências anteroposterior e de perfil (C) de ulnas radiopacas após a fixação da osteotomia com o parafuso intramedular.

Após a redução da osteotomia utilizando pinça óssea tipo Weber, foi introduzido um fio de Kirshner (2,0 mm) para auxiliar no controle rotacional do fragmento durante a montagem. O ponto de entrada do fio-guia no olécrano foi estrategicamente posicionado para criar um trajeto desviado do eixo central do osso, compensando o varo fisiológico da ulna. Com o fio-guia posicionado, o ponto de entrada para o parafuso no olécrano foi perfurado com uma broca canulada (3,2 mm). A fixação da osteotomia foi realizada com a inserção longitudinal no canal medular de um parafuso canulado (7,0 × 9,0 mm) com rosca parcial e arruela ( Fig. 1 ).

#### Parafusos Transcorticais

**Fig. 2:**
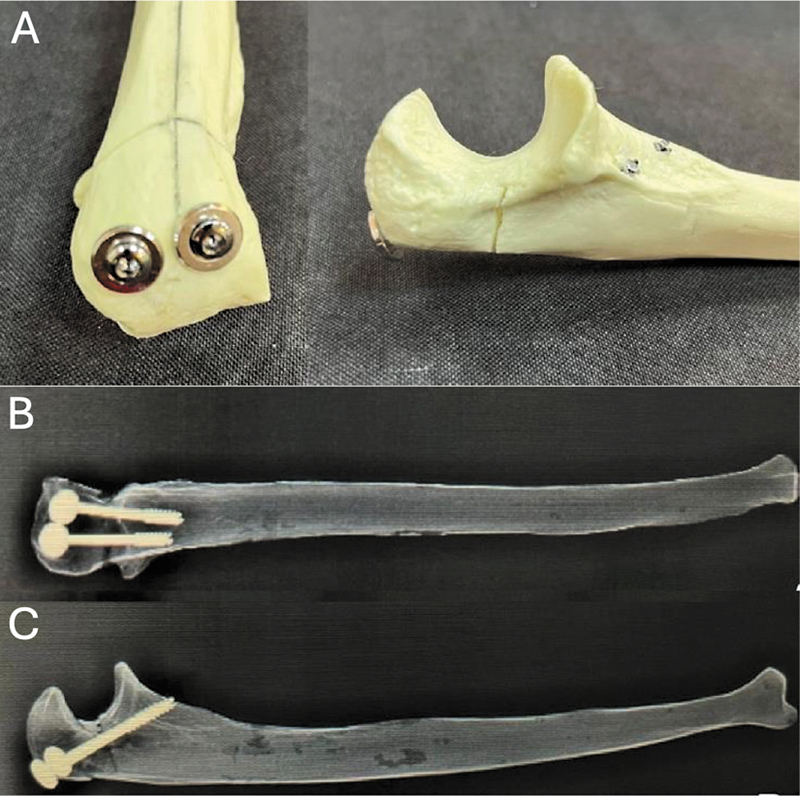
Fixação com parafusos transcorticais (TC). (A) Fixação da osteotomia realizada pela inserção perpendicular de dois parafusos transcorticais. (B) Radiografias nas incidências anteroposterior e de perfil (C) de ulnas radiopacas após a fixação da osteotomia com parafusos transcorticais.

Após a redução da osteotomia, foram inseridos dois fios-guias paralelos na região proximal do olécrano, atravessando a área da osteotomia e preservando a região articular da ulna. A distância entre os fios foi mantida entre 10 e 12 mm ao longo do percurso, com o ponto de saída localizado imediatamente anterior ao processo coronoide. Através dos fios-guias, foram realizados orifícios transcorticais com broca canulada (2,5 mm). Após a fresagem dos orifícios e da medição do tamanho do canal, a fixação da osteotomia foi obtida por meio da inserção perpendicular de 2 parafusos (4 × 42–26 mm) com rosca parcial e arruela ( Fig. 2 ).

### Testes Biomecânicos

**Fig. 3:**
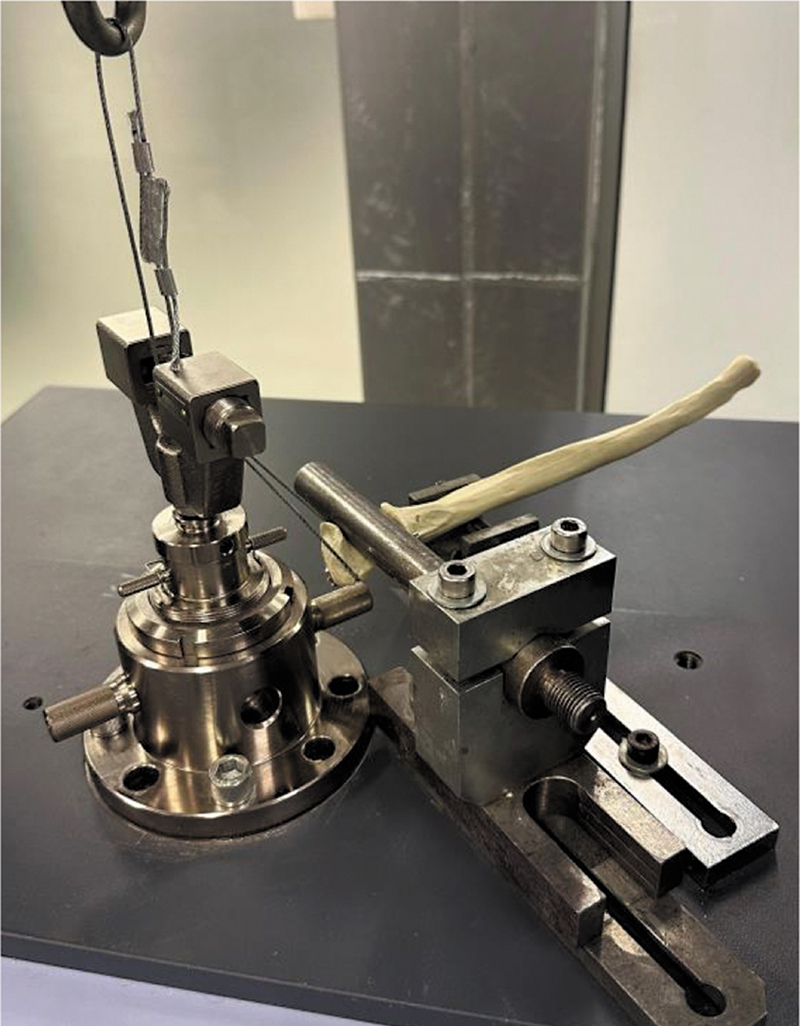
Avaliação biomecânica dos sistemas de fixação com os corpos de prova montados em máquina universal de ensaios SHIMADZU AGS-X.

As características biomecânicas dos sistemas de fixação foram avaliadas por protocolo que associa ensaio com carregamento cíclico durante um número pré-determinado de ciclos seguido de ensaio monotônico de tração até o limite de resistência mecânica de cada sistema. Os experimentos foram realizados em uma máquina universal de ensaios SHIMADZU AGS-X equipada com célula de carga Shimadzu e capacidade de 100 kN (Shimadzu Corporation). Os corpos de prova foram posicionados na máquina de ensaios utilizando um dispositivo de fixação composto por: (1) uma base de apoio fixada na plataforma da máquina, que serviu como ponto de reação para a força aplicada ao fragmento reconstruído; e (2) uma haste de reação horizontal, inserida na cavidade articular da ulna, simulando a resistência oferecida pela tróclea umeral. Para reproduzir a ação do tendão do tríceps, a tração foi aplicada ao olécrano (fragmento ósseo) por meio de um cabo de aço ( Fig. 3 ).

**Fig. 4:**
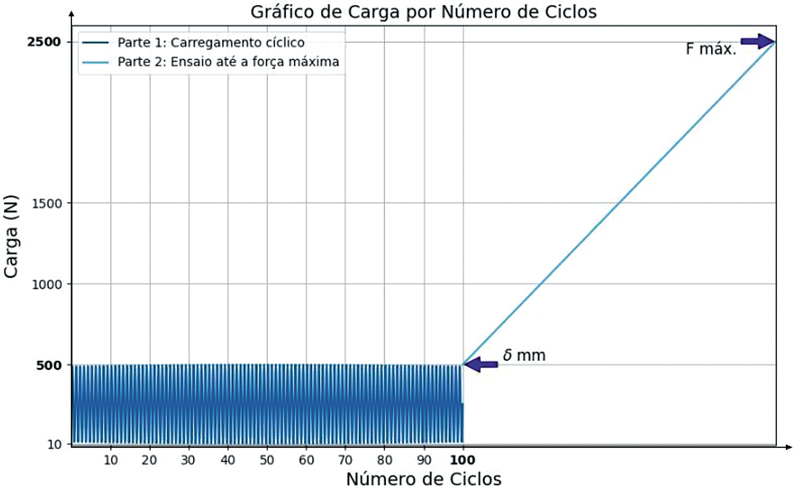
Representação gráfica da carga aplicada sobre os corpos de prova por ciclos de carregamento.

Após a montagem, foi aplicada uma pré-carga de 10 N, seguida de 100 ciclos de carregamento entre 10 N e 500 N. Após a conclusão dos 100 ciclos, a carga de 500 N foi mantida e o *gap* na região da osteotomia foi medido com régua calibradora. O afastamento da osteotomia em > 2 mm foi utilizado como critério de falha. Após a medição, foi aplicado carregamento monotônico de tração com controle de deslocamento (10 mm/min) até atingir a falha da fixação, determinando-se a força de resistência máxima e o modo de falha associado ( Fig. 4 ). A rigidez das 2 montagens foi medida através do cálculo da inclinação da curva força x deslocamento na região entre 520 e 700 N.

### Análise Estatística

Para comparar as médias das variáveis obtidas nos testes dinâmicos e a carga média de falha definida no teste estático foram utilizados o teste de análise de variância (ANOVA, do inglês *analysis of variance* ) unidirecional e o teste t de Student, assumindo variâncias diferentes para as aberturas das osteotomias e iguais para a força máxima. A significância estatística foi definida para valores de *p* < 0,05. A análise estatística foi realizada com o RStudio, versão 2024.04.2 + 764, e a linguagem R, versão 4.4.1 (Posit PBC).

## Resultados

### Teste de Carregamentos Cíclicos e Quase-Estáticos

**Fig. 5:**
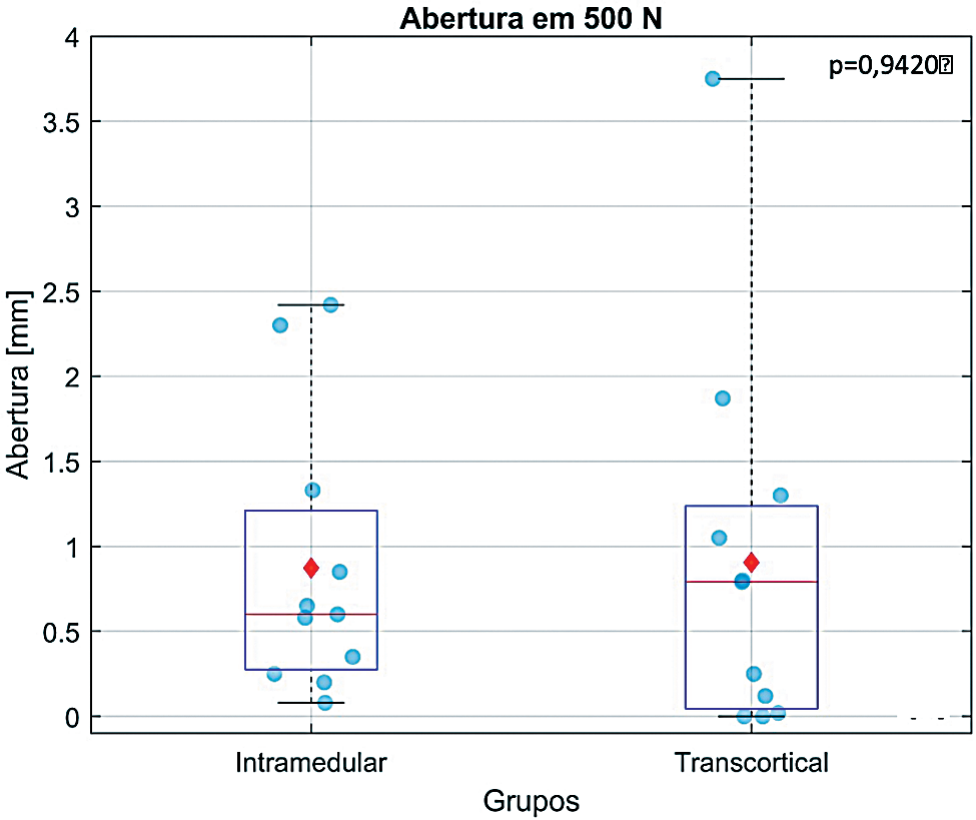
Teste de carregamento cíclico (dinâmico). Gráfico *Box plot* mostrando a abertura das osteotomias nos dois métodos de fixação após 100 ciclos com carga de 500 N. Não foi observada falha em nenhuma das montagens (gap < 2mm). Os valores estão representados pela mediana (linha vermelha), média (ponto vermelho) e percentis 25% e 75% (linhas azuis).

Nenhum dos construtos apresentou falha > 2 mm após a aplicação da carga quase-estática de 500 N, sem diferença significativa nos valores dos *gaps* entre os dois grupos ( *p* = 0,9420). Os valores médios de abertura das osteotomias estão resumidos na Tabela 1 e ilustrados na Fig. 5 .

**Tabela 1:** Resultados dos testes de carregamentos cíclico e quase-estáticos e de resistência máxima

	Tipo de parafuso		
	*Intramedular*	*Transcortical*	Valor-p*
Abertura da osteotomia em 500 N (mm)	0,87 ± 0,81	0,90 ± 1,13	0,9420
Força máxima antes da falha (N)	1641,18 ± 304,08	1293,65 ± 442,30	0,0459
Rigidez (N/mm)	264,65 ± 38,23	258,99 ± 20,72	0,6704

**Notas** : Valores expressos como média ± desvio padrão. * Teste *t* de Student.

### Teste de Resistência Máxima

**Fig. 6:**
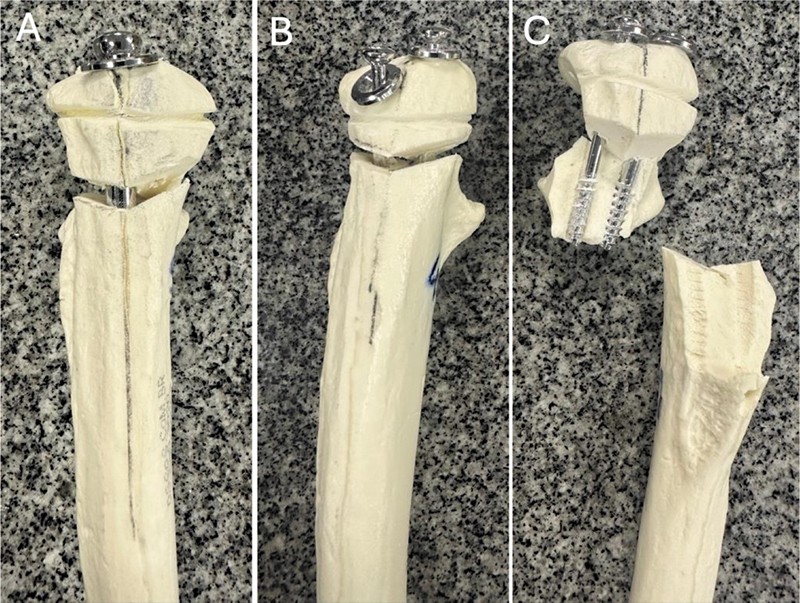
Modos de falha da fixação após carregamento monotônico de tração para determinação da força máxima de rompimento. (A) Abertura da osteotomia em > 2 mm na fixação com parafuso IM ou (B) parafusos TC. (C) Soltura e fratura dos fragmentos na fixação com parafusos TC.

**Fig. 7:**
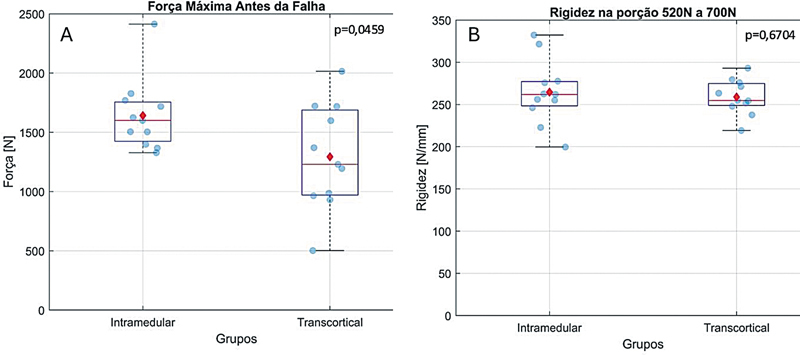
Teste de carregamento monotônico (estático). Gráfico *Box plot* mostrando a força máxima antes da falha e a rigidez dos dois métodos de fixação da osteotomia. (A) A carga de falha no grupo IM foi 1,27 vezes maior do que na fixação com parafusos transcorticais ( *p* = 0,0459). (B) Na região entre 520 N e 700 N não foi observada diferença entre a rigidez dos dois sistemas de fixação ( *p* = 0,6704). Os valores estão representados pela mediana (linha vermelha), média (ponto vermelho) e percentis 25% e 75% (linhas azuis).

Após a aplicação de carregamento monotônico de tração com controle de deslocamento com velocidade de 10 mm/min, todos os espécimes falharam devido ao afastamento da osteotomia em > 2 mm. A força máxima de falha no grupo IM foi 1,27 vezes maior do que no grupo TC ( *p* = 0,0459). No grupo IM, as falhas ocorreram principalmente pelo afastamento dos fragmentos em direções opostas ( Fig. 6A ). No grupo TC, as falhas resultaram do afastamento da osteotomia ( Fig. 6B ) e por fratura dos fragmentos ( Fig. 6C ). Não foi observada diferença significativa entre a rigidez da fixação com TC ou IM ( *p* = 0,67). Os valores médios da força máxima na falha e da rigidez das montagens estão descritos na Tabela 1 e na Fig. 7 .

## Discussão

No presente estudo, utilizamos ossos compósitos de ulna para comparar as propriedades biomecânicas da fixação da osteotomia do olécrano tipo Chevron, utilizando um parafuso IM ou dois parafusos TC. Após a realização de 100 ciclos de carga de tração, entre 10 N e 500 N, nenhuma das 2 montagens apresentou falha. Estes resultados sugerem que ambos os métodos de fixação são igualmente eficazes em suportar cargas cíclicas dentro dessa faixa de demanda mecânica, sem comprometer a estabilidade articular. No teste de carregamento monotônico, a força máxima de falha no grupo IM foi 1,27 vezes maior do que no grupo TC, evidenciando uma maior capacidade do sistema IM em suportar cargas antes da falha, o que sugere uma vantagem em termos de resistência mecânica.

Diversos estudos clínicos e biomecânicos têm abordado as vantagens e complicações associadas às diferentes técnicas de fixação das fraturas do úmero distal. 4 6 12 13 17 20 As técnicas que envolvem a osteotomia do olécrano são amplamente utilizadas para a redução aberta e fixação interna de fraturas intra-articulares, por oferecerem melhor visualização da fratura. 4 5 Entre essas técnicas, a osteotomia do tipo Chevron é a mais comumente empregada, devido à sua capacidade de proporcionar maior estabilidade rotacional e uma área de contato ampliada entre as superfícies ósseas, otimizando as condições locais para a consolidação óssea. 4 7 9 11 No entanto, complicações como falha de consolidação, perda da redução e implante sintomático podem ocorrer, 5 12 13 14 o que torna a escolha das técnicas de fixação um tema recorrente na literatura. 4 6 12 13

Vários estudos investigaram a estabilidade das técnicas de fixação de osteotomias do olécrano, tanto com parafusos TC 10 21 22 quanto com parafuso IM. 15 17 23 Wagener et al. 7 realizaram um dos poucos estudos biomecânicos comparando técnicas de fixação da osteotomia do tipo Chevron, utilizando ossos cadavéricos. Neste estudo, as osteotomias foram fixadas com fios de Kirschner TC associados a bandas de tensão e parafusos, com ou sem bandas de tensão. Os ensaios biomecânicos foram realizados aplicando forças entre 200 e 500 N. Os resultados indicaram que a fixação apenas com parafuso apresentava rotação e translação da porção proximal da osteotomia quando submetida a forças > 350 N, enquanto a combinação de parafuso com banda de tensão aumentava significativamente a capacidade da osteotomia de suportar maiores forças aplicadas ao tríceps. 7 Outro estudo biomecânico, também realizado em ossos cadavéricos, demonstrou que a fixação da osteotomia com parafusos de compressão associados a bandas de tensão apresentou melhor desempenho. 20

No contexto clínico, a maioria dos estudos combina, na mesma casuística, os resultados da fixação de fraturas e osteotomias, utilizando uma variedade de técnicas cirúrgicas, o que dificulta a análise crítica do desempenho mecânico de cada método. O primeiro estudo a relatar os desfechos associados à fixação da osteotomia do olécrano com dois parafusos transcorticais foi conduzido por Dumartinet-Gibaud et al. 10 Neste estudo retrospectivo com 39 pacientes, a fixação com 2 parafusos TCs demonstrou melhores resultados clínicos e radiológicos, além de uma menor taxa de revisões cirúrgicas (21%) em comparação com a fixação com banda de tensão e fio de Kirschner (56%). Os autores também observaram menor taxa de perda de fixação (7% versus 24% com banda de tensão). 10 Em outro estudo retrospectivo, Gill et al. 21 relataram os resultados de 27 casos, incluindo 17 fraturas e 10 osteotomias do olécrano, todas fixadas com 2 parafusos TCs. Não houve perda de redução ou necessidade de revisão das osteotomias, e os autores destacaram a segurança, simplicidade e baixos índices de complicações da técnica. 21

Uma alternativa à fixação com dois parafusos TC é a fixação com um parafuso IM. Um estudo biomecânico comparando a fixação da osteotomia com parafuso IM ou placa não encontrou diferenças significativas na intensidade de carga até a falha. 15 Cañete San Pastor et al. 17 avaliaram retrospectivamente 26 pacientes com fratura supraintercondilar do úmero distal, submetidos à fixação da osteotomia com um parafuso canulado IM. Após 1 ano de acompanhamento, todas as osteotomias apresentaram consolidação radiológica, com tempo médio de 112 dias, confirmando a eficácia e a possível superioridade da técnica em relação a outros métodos estabelecidos na literatura. 17 Resultados semelhantes foram relatados por Meldrum et al. 24 em um estudo retrospectivo com 92 pacientes, onde 37% necessitaram de remoção dos implantes, enquanto nenhum dos 10 pacientes com fixação por parafuso IM necessitou remover o implante. Ocalan et al. 23 também observaram menores índices de remoção de implantes em osteotomias fixadas com parafuso IM (18%) em comparação com a fixação com placa (75%).

Identificamos algumas limitações em nosso estudo que merecem ser destacadas. Em estudos biomecânicos, os resultados obtidos com tecido cadavérico geralmente apresentam maior poder translacional do que aqueles obtidos com materiais artificiais. Optamos por usar ulnas artificiais de espuma de poliuretano para garantir a homogeneidade da amostra, minimizando variações que poderiam influenciar negativamente os resultados. Ulnas humanas apresentam variações em tamanho e grau de osteopenia, o que pode dificultar a reprodução exata dos resultados. Por outro lado, ulnas artificiais permitem maior controle sobre os parâmetros analisados no estudo.

## Conclusão

Os resultados mostraram que ambas as técnicas de fixação proporcionaram rigidez semelhante e estabilidade cíclica eficaz até 500 N, preservando a integridade da osteotomia. A fixação com parafuso IM apresentou maior resistência à carga máxima antes da falha, sugerindo vantagem em situações de maior exigência mecânica. Os achados estão limitados ao modelo biomecânico utilizado, mas podem orientar a escolha clínica da técnica e futuras pesquisas sobre a osteotomia tipo Chevron.
